# Near-Infrared Photobiomodulation of the Peripheral Nerve Inhibits the Neuronal Firing in a Rat Spinal Dorsal Horn Evoked by Mechanical Stimulation

**DOI:** 10.3390/ijms24032352

**Published:** 2023-01-25

**Authors:** Daisuke Uta, Naoya Ishibashi, Takahiro Konno, Yuki Okada, Yuki Kawase, Shinichi Tao, Toshiaki Kume

**Affiliations:** 1 Department of Applied Pharmacology, Faculty of Pharmaceutical Sciences, University of Toyama, Toyama 930-0194, Japan; 2 Department of Applied Pharmacology, Graduate School of Medicine and Pharmaceutical Sciences, University of Toyama, Toyama 930-0194, Japan; 3 Biomedical Engineering Laboratories, Teijin Institute for Bio-Medical Research, Teijin Pharma Ltd., Tokyo 191-8512, Japan; 4 Toxicology & DMPK Research Department, Teijin Institute for Bio-Medical Research, Teijin Pharma Ltd., Tokyo 191-8512, Japan

**Keywords:** in vivo extracellular recording, spinal dorsal horn, lamina II, pain, peripheral nerve, photobiomodulation, low-level laser therapy

## Abstract

Photobiomodulation has analgesic effects via inhibition of nerve activity, but few reports have examined the effects on the spinal dorsal horn, the entry point for nociceptive information in the central nervous system. In this study, we evaluated the effects of laser irradiation of peripheral nerve axons, which are conduction pathways for nociceptive stimuli, on the neuronal firing in lamina II of the spinal dorsal horn of a rat evoked by mechanical stimulation with von Frey filaments (vFF). In order to record neuronal firing, electrodes were inserted into lamina II of the exposed rat spinal dorsal horn. The exposed sciatic nerve axons were irradiated with an 808 nm laser. The 26.0 g vFF-evoked firing frequency was inhibited from 5 min after laser irradiation and persisted for 3 h. Sham irradiation did not alter the firing frequency. Laser irradiation selectively inhibited 15.0 and 26.0 g vFF-evoked firing, which corresponded to nociceptive stimuli. Histopathological evaluation revealed no damage to the sciatic nerve due to laser irradiation. These results indicate that neuronal firing is inhibited in lamina II of the spinal dorsal horn, suggesting that laser irradiation inhibits Aδ and/or C fibers that conduct nociceptive stimuli.

## 1. Introduction

Photobiomodulation (PBM) or low-level laser/light therapy is a treatment method that uses a laser or a light-emitting diode and has a variety of effects [1]. PBM is mainly used for analgesic treatment [2] and is effective for both acute and chronic pain [3,4]. Meta-analyses have validated the efficacy of PBM in relieving clinically significant pain in many common diseases, including chronic neck pain [5], headache [6], postoperative pain [7], and tendinopathy [8]. In addition, PBM has few side effects [5,9] and is expected to be a complementary or alternative treatment to pharmaceutical-based therapies.

One of the challenges of PBM research is that the mechanism of its analgesic effect is poorly understood [10]. Previous reports have described anti-inflammatory effects [11], activation of peripheral opioid receptors [12], microtubule aggregation [13,14,15], and nerve conduction block [16,17,18] as the mechanism of action. Several reports have used in vivo electrophysiological techniques that can evaluate the analgesic effect in detail to investigate how PBM blocks nerve conduction [16,17,18]. These studies suggest that PBM may inhibit Aδ and C fibers, many of which function as nociceptors.

However, few studies have verified the effect of PBM in the spinal dorsal horn. The spinal dorsal horn is the entry point for nociceptive information in the central nervous system [19]. Aδ and C fibers terminate on the second layer (lamina II) of the dorsal horn [19], and via interneurons in the spinal cord, signals are transmitted to the brain. Therefore, we hypothesized that PBM using a laser on the peripheral nerve inhibits the neuronal firing in lamina II of the dorsal horn evoked by noxious stimulation. To the best of our knowledge, only two studies have examined neural activity in the spinal dorsal horn [20,21]. However, these reports do not mention from which layer of the spinal dorsal horn the recordings were taken, making it difficult to conclude that nociceptive stimulus-evoked neural activity via Aδ and C fibers was inhibited in the spinal dorsal horn. Recording neuronal firing in lamina II of the spinal dorsal horn [22] could reveal whether nerve conduction through the Aδ and C fibers ascending from the periphery to the central nervous system is also blocked following laser radiation delivery onto the peripheral nerve.

The aim of this study was to examine the effects of laser irradiation of the sciatic nerve on the neuronal firing in lamina II of the dorsal horn evoked by mechanical stimulation of the cutaneous receptive field.

## 2. Results

### 2.1. Effects of Photobiomodulation on the Neuronal Firing in Lamina II of Spinal Dorsal Horn Neurons Evoked by Mechanical Stimulation

**Figure 1:**
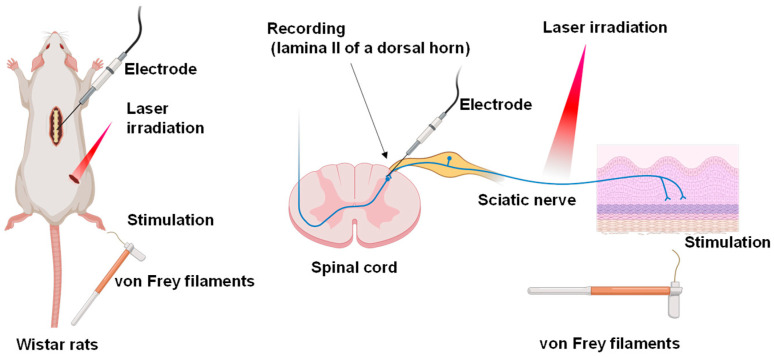
Schematic diagram of our experimental setup. Electrodes were inserted into lamina II of a rat spinal dorsal horn for recording. The skin of the right hind leg was incised to expose the sciatic nerve and irradiated with a laser. Mechanical stimulation with von Frey filaments (vFF) was applied to the cutaneous receptive field.

We examined the effects of laser irradiation on the neuronal transmission evoked by mechanical stimulation of the skin using extracellular recordings from spinal dorsal horn neurons (Figure 1).

**Figure 2:**
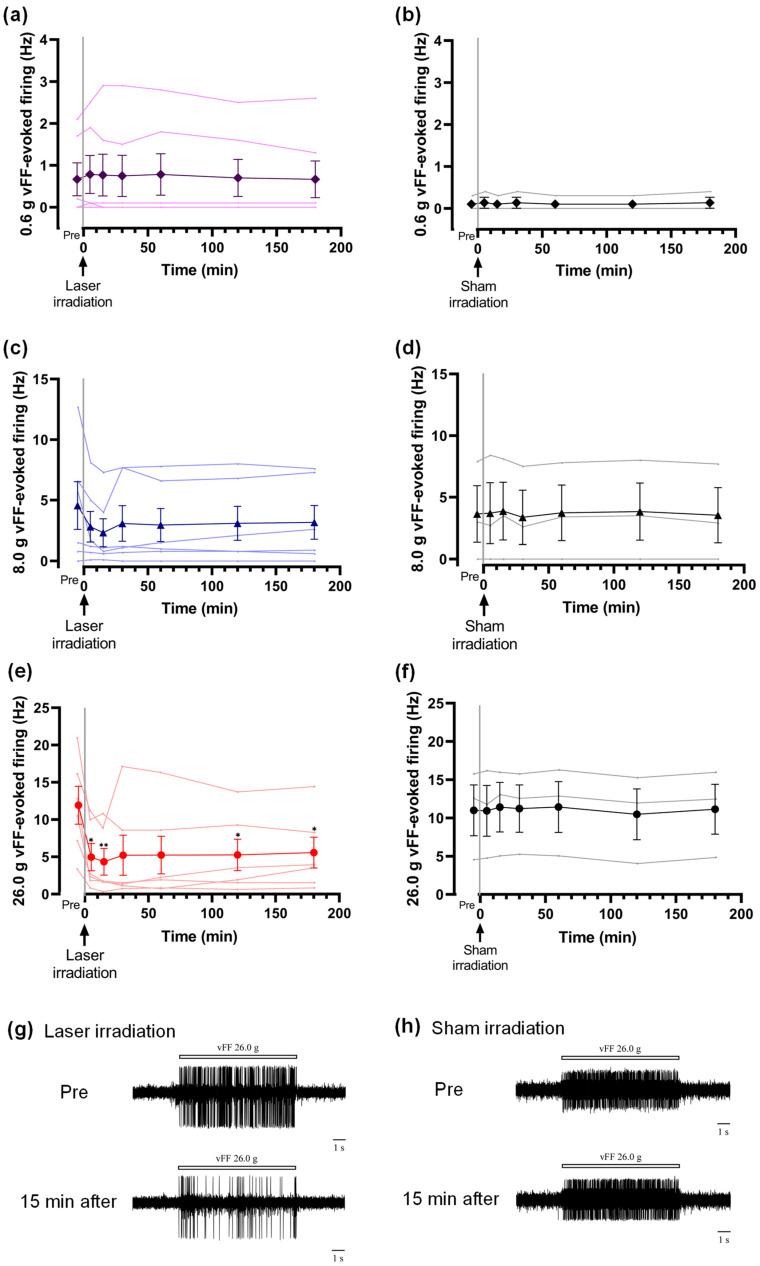
Effects of laser irradiation on the neuronal firing in lamina II of the spinal dorsal horn evoked by mechanical stimulation. (**a**) Laser irradiation did not affect the 0.6 g vFF-evoked firing. (**b**) Sham irradiation did not change the 0.6 g vFF-evoked firing. (**c**) Laser irradiation did not significantly inhibit the 8.0 g vFF-evoked firing. (**d**) Sham irradiation did not affect the 8.0 g vFF-evoked firing. (**e**) Laser irradiation of the sciatic nerve significantly inhibited the 26.0 g vFF-evoked firing frequency. The effect lasted for 3 h. (**f**) Sham irradiation did not affect the 26.0 g vFF-evoked firing. (**g**) Representative trace of the 26.0 g vFF-evoked firing recorded in (**e**) before sham irradiation (Pre) and 15 min after the irradiation. The vFF stimulation was applied for 10 s. (**h**) Representative trace of the 26.0 g vFF-evoked firing recorded in (**f**) before sham irradiation (Pre) and 15 min after. The data are presented as the means ± SEM; * *p* < 0.05 and ** *p* < 0.01 vs. Pre using Dunnett’s multiple comparison test (*n* = 3–6); vFF, von Frey filaments; SEM, standard error of the mean.

Laser irradiation had no effect on the 0.6 and 8.0 g vFF-evoked firing frequency (Figure 2a,c). Laser irradiation significantly reduced the 26.0 g vFF-evoked firing frequency. The decrease in firing frequency was observed 5 min after the irradiation and continued for 3 h (Figure 2e,g). Sham irradiation (only the placement of a laser irradiation probe) had no effect on vFF-evoked firing frequency (Figure 2b,d,f,h).

**Figure 3:**
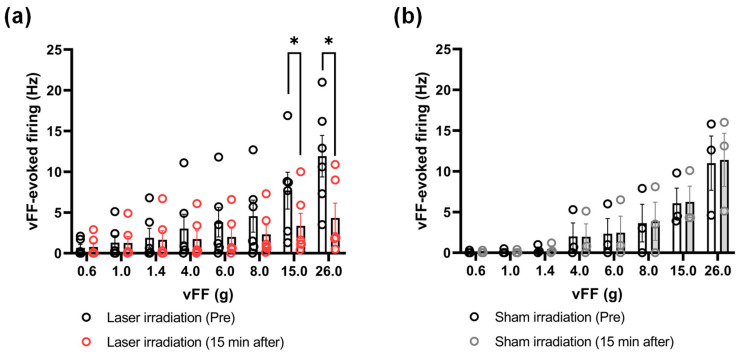
Comparison of frequencies among the 0.6, 1.0, 1.4, 4.0, 6.0, 8.0, 15.0, and 26.0 g vFF-evoked firing at 15 min after laser irradiation, when the 26.0 g vFF-evoked firing was the most inhibited. (**a**) The 15.0 and 26.0 g vFF-evoked firing was significantly inhibited while there were no significant differences in the 0.6, 1.0, 1.4, 4.0, 6.0, and 8.0 g vFF-evoked firing. (**b**) There were no significant differences for all vFFs after sham irradiation. The data are presented as the means ± SEM; * *p* < 0.05 vs. Pre using Wilcoxon matched-pairs signed-rank test (*n* = 3–6); vFF, von Frey filaments; SEM, standard error of the mean.

The frequency of the 0.6, 1.0, 1.4, 4.0, 6.0, 8.0, 15.0, and 26.0 g vFF-evoked firing was compared at 15 min after laser irradiation, when the frequency of the 26.0 g vFF-evoked firing was most inhibited (Figure 3a). In addition to 26.0 g, the 15.0 g vFF-evoked firing frequency was also significantly inhibited. No significant differences were identified among 0.6, 1.0, 1.4, 4.0, 6.0, and 8.0 g vFF-evoked firing. Sham irradiation showed no change in all the filaments (Figure 3b).

### 2.2. Effects of Lidocaine on the Neuronal Firing in Lamina II of Spinal Dorsal Horn Neurons Evoked by Mechanical Stimulation

**Figure 4:**
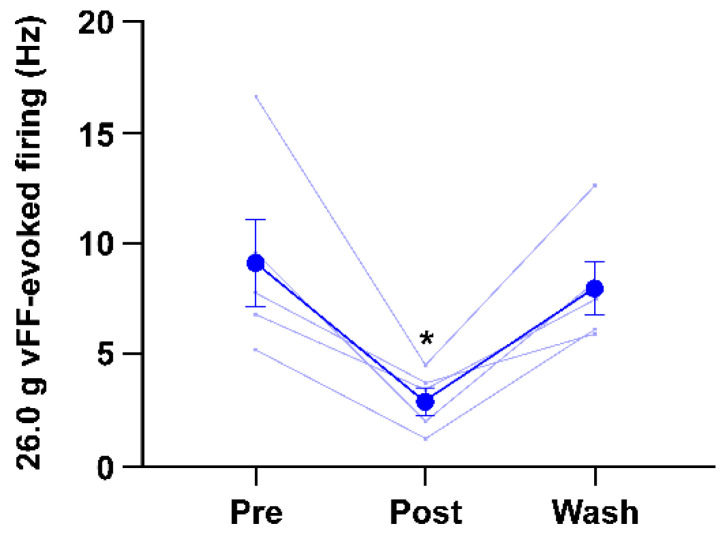
Administration of 0.5% lidocaine to the vFF stimulation site significantly inhibited the 26.0 g vFF-evoked firing frequency. After the wash, the firing frequency returned to the initial level. The data are presented as the means ± SEM; * *p* < 0.05 vs. Pre using Dunnett’s multiple comparison test (*n* = 5); vFF, von Frey filaments; SEM, standard error of the mean.

Administration of 0.5% lidocaine used as a local anesthetic to the vFF stimulation site significantly inhibited the 26.0 g vFF-evoked firing frequency in lamina II of the spinal dorsal horn (Figure 4).

**Figure 5:**
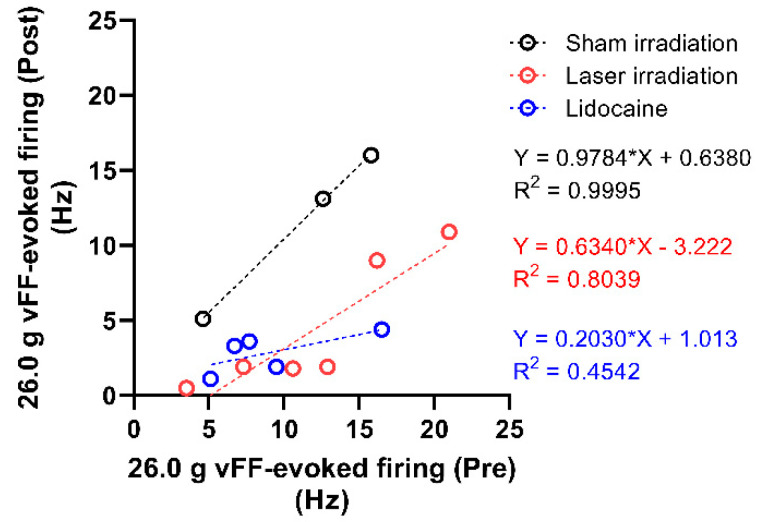
Correlation between the 26.0 g vFF-evoked firing before and after intervention for laser irradiation, sham irradiation, and lidocaine administration, respectively. For laser and sham irradiation, the values on the vertical axis depict the values recorded 15 min after irradiation. For lidocaine, the vertical axis depicts the values recorded 5 min after the administration. Each dot in the figure is shown as a response of an individual neuron; vFF, von Frey filaments.

Correlations of the 26.0 g vFF-evoked firing frequency before and after intervention were compared between sham irradiation, laser irradiation, and lidocaine (Figure 5). The correlation coefficients were r = 0.9998, 0.8966 (95% confidence interval (CI): 0.312–0.9887) and 0.6739 (95% CI: −0.5139–0.9759) for sham irradiation, laser irradiation, and lidocaine, respectively. The degree of decrease in the correlation coefficient from 1.000 corresponds to the effect of the intervention. The slopes were 0.9784, 0.6340, and 0.2030 for sham irradiation, laser irradiation, and lidocaine, respectively (Figure 5).

### 2.3. Histopathological Evaluation of the Sciatic Nerve

**Figure 6:**
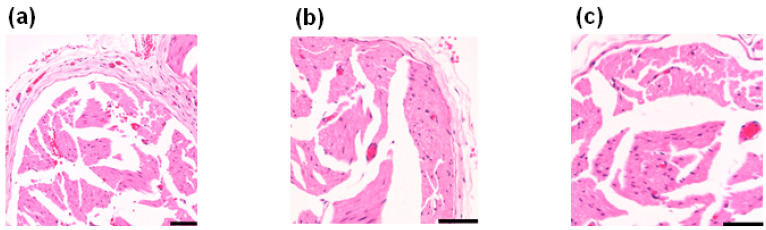
Bilateral sciatic nerves of the sham- and laser-irradiated rats were evaluated for pathology by HE staining (*n* = 3 each). There were no differences in findings between all the conditions, and no histopathological changes such as nerve damage due to laser irradiation were observed. (**a**) Representative image of the left sciatic nerve of sham-irradiated rats (intact). (**b**) Representative image of the right sciatic nerve of sham-irradiated rats (nerve exposed). (**c**) Representative image of the right sciatic nerve of laser-irradiated rats (nerve exposed). Black bars: 50 µm.

In order to examine if laser irradiation damaged the sciatic nerve, the sciatic nerve was harvested after laser or sham irradiation and evaluated histopathologically by hematoxylin and eosin (HE) staining (Figure 6). Both sides of the sciatic nerve were sampled, including the sciatic nerve on the opposite side (left side) where no skin incision or laser irradiation was applied. There was no difference in findings with or without laser irradiation or skin incision, suggesting that laser irradiation did not cause histopathologic changes.

## 3. Discussion

In this study, we demonstrated that 808 nm laser irradiation of the sciatic nerve inhibited the neuronal firing in lamina II of the dorsal horn evoked by mechanical stimulation without damaging the sciatic nerve in anesthetized rats. Our study is the first to demonstrate that blocking of conduction by laser irradiation occurred in lamina II of the dorsal horn.

We used vFFs of different thicknesses to quantitatively compare the effect of mechanical stimulation intensity on the inhibitory effect of laser irradiation (Figure 3). At 15 min after laser irradiation, significant differences were observed for vFFs of 15.0 and 26.0 g, but not for vFFs of 8.0 g or less. In behavioral experiments of pain, 15.0 or 26.0 g can be considered a nociceptive stimulus, 0.6 g—a non-nociceptive stimulus, and 4.0 or 6.0 g—an intermediate stimulus [22]. Therefore, it is suggested that the laser irradiation inhibited nociceptive stimuli. Similar to our report, Tsuchiya et al. reported that 830 nm lasers inhibit the neural activity evoked by pinch stimuli, which are considered nociceptive, but not by brush stimuli, which are considered non-nociceptive [17]. Since nociceptive stimuli are transmitted through Aδ and C fibers [19], our results are more supportive of the mechanism of action that laser irradiation blocks Aδ and/or C fibers.

In the previous electrophysiological studies examining the conduction blockage in the spinal cord by laser irradiation, Shimoyama et al. reported percutaneous He–Ne laser irradiation of the peroneal nerve for 30 min suppressed the neuronal firing for 15 min in the rat spinal dorsal horn induced by formalin administration to the peripheral skin [20]. In addition, Kono et al. showed that the cord dorsum potential evoked by electrical stimulation of the peripheral sural nerve axons in cats was suppressed during 10 min of laser irradiation of the axons and returned to the same level as before after laser irradiation [21]. These two reports showed different results: one showed inhibition of nociception-evoked neural activity after laser irradiation [20], while the other showed inhibition of neural activity during laser irradiation and not after [21]. Furthermore, there is no description of the spinal layer for recordings [20,21]; thus, it is unclear whether the laser irradiation inhibited nerve conduction by Aδ and/or C fibers in the dorsal horn. Our results showed that the nociceptive stimulation-evoked neuronal firing in lamina II was inhibited after laser irradiation, thus supporting the report of Shimoyama et al. [20] and suggesting that laser irradiation inhibited Aδ and/or C fibers. 

Recently, Yan et al. reported that percutaneous laser irradiation of four different locations on the sciatic nerve caused a decrease in the amplitude of short latent somatosensory evoked potentials (SSEPs) 10 min after laser irradiation in the 650 nm condition and 10 and 20 min in the 808 nm condition, as well as increased the latency of compound muscle action potentials [18]. Chow et al. from the same group [23] used the same laser wavelength and power density as in the previous study [18] and percutaneously irradiated a single location on the sciatic nerve for the same or four times the duration as in the previous study [18]. They showed that the SSEP amplitude increased only at 808 nm and with four times the duration but did not change under other conditions [23]. Therefore, even if the amount of total energy density is the same, the effect on neural activity may change with the number of laser irradiations and irradiation sites. In contrast to these reports, our study showed that laser irradiation of a single site on the sciatic nerve inhibited nociceptive stimulus-evoked neuronal firing. This difference may be due to the intensity of the laser on the nerve being higher than that of the studies of Yan et al. [18] and Chow et al. [23] as we directly applied the laser on the sciatic nerve. A previous study showed that laser power density at 808 nm decreased to less than 10% of the skin surface when it penetrated about 1 cm of skin in rats [24]. Further studies are needed that examine the effects of transcutaneous and low-intensity laser irradiation in our experimental setup.

Histopathological evaluation by HE staining showed that direct laser irradiation at 1 W/cm^2^ and 180 J/cm^2^ did not damage the sciatic nerve, suggesting that the inhibition of neuronal firing was not due to an injury of the sciatic nerve by laser irradiation (Figure 6). PBM treatment has few side effects [9], for example, for neck pain [5] and stellate ganglion block [25], with no adverse events reported in each meta-analysis. Our data showing suppression of firing without producing nerve damage support these studies.

Administration of local anesthetic lidocaine to the vFF stimulation site significantly inhibited the 26.0 g vFF-evoked firing frequency in the spinal dorsal horn (Figure 4). In the correlation analysis, the slope of the approximate curve of the scatter plot was smaller for lidocaine than for the laser-irradiated group (Figure 5). This suggests that the inhibition of neuronal firing by laser irradiation may be inferior to the effects of lidocaine. However, Holanda et al. compared radiofrequency treatment, laser irradiation, and lidocaine in patients with lower back pain and reported that laser irradiation and lidocaine showed more analgesic effects than radiofrequency treatment, and laser irradiation and lidocaine showed similar effects [26]. When comparing laser irradiation and lidocaine, one of the characteristics of laser irradiation is its noninvasiveness [5,9], while lidocaine administration by injection could be painful and invasive. Another advantage of a laser is that it has the potential to selectively treat pain without changing tactile sensations while lidocaine can inhibit tactile sensations and the motor function depending on its concentration [27]. A detailed comparison of the efficacy of analgesic drugs such as lidocaine and PBM may clarify the usefulness and applications of PBM and contribute to the elucidation of the mechanism.

This study had several limitations. First, since we used healthy rats, it is not clear whether neuronal firing in hyperalgesia and allodynia is also inhibited. The effects of lasers on hyperalgesia and allodynia can be evaluated using animal models such as neuropathic pain models. PBM has been used, for example, to treat musculoskeletal pain [28], but there is currently a lack of basic knowledge about its therapeutic mechanism. A basic study of PBM may be helpful in positioning PBM in the context of widely used drug therapies and when considering appropriate usage. Second, we exposed the sciatic nerve and directly irradiated it with a laser. Although the nerve was exposed in order to ensure that the laser directly irradiated the nerve, laser irradiation is delivered percutaneously in clinical practice. A future study can evaluate whether percutaneous irradiation also inhibits firing in lamina II of the spinal dorsal horn. Third, the duration of the inhibitory effect of lasers differs between humans and animals. As mentioned above, the maximum time that can be measured with our experimental system is approximately 3 h. In a report using rodents, the effects of the laser disappeared 24 h [15] and 48 h [18] after laser irradiation in healthy animals. In clinical practice, a carryover effect, in which the analgesic effect accumulates, has been reported. The effect persists for several weeks after stopping laser treatment [5]. To our knowledge, there are no reports in animals regarding the carryover effect of laser analgesia. If the same carryover effect as in the clinical setting is observed in animal models of pain by multiple laser irradiations, the molecular mechanism of the carryover effect may be elucidated.

## 4. Materials and Methods

All the experiments were performed in accordance with the Guiding Principles for Care and Use of Animals in the Field of Physiological Sciences of the Physiological Society of Japan and approved by the local Animal Experiment Committees of the University of Toyama (approval No. A2020PHA-12 (9 April 2020)). All efforts were made to minimize animal suffering and the number of animals used for the studies.

### 4.1. Animals

Seven-week-old Wistar rats (Japan SLC Corporation, Hamamatsu, Japan) were used for the electrophysiology experiments. The Wistar rats were kept under environmental control with a 12 h light/dark cycle (lights on at 7:00 AM), temperature (permissive range) of 23 °C (20–26 °C), and humidity (permissive range) of 55% (30–60%), with free access to food and water.

### 4.2. In Vivo Preparation and Extracellular Recordings from Lamina II Neurons

The methods used for the in vivo extracellular recordings are described in detail elsewhere [29]. Briefly, the adult male Wistar rats were anesthetized with urethane (1.2–1.5 g/kg, intraperitoneal administration). Urethane has minimal effects on the cardiovascular and respiratory systems and maintains a stable level of anesthesia for an extended period [30]. Urethane also does not require additional dosing, except in a few cases. Importantly, no additional doses were administered in our experiment. A thoracolumbar laminectomy was performed, exposing the levels from Th11 to L4, and the animal was then placed in a stereotaxic apparatus. After removing the dura and cutting the arachnoid membrane to create a window large enough to accommodate a tungsten microelectrode, the surface of the spinal cord was irrigated with a 95% O_2_–5% CO_2_ equilibrated Krebs solution (10–15 mL/min) containing the following (in mM): 117 NaCl, 3.6 KCl, 2.5 CaCl_2_, 1.2 MgCl_2_, 1.2 NaH_2_PO_4_, 11 glucose, and 25 NaHCO_3_, through glass pipettes at 37 ± 1 °C.

### 4.3. In Vivo Extracellular Recordings from Lamina II Neurons

The methods used for the in vivo extracellular recordings are described in detail elsewhere [31]. Briefly, extracellular single-unit recordings of the superficial dorsal horn (lamina II) neurons were performed as follows: recordings were conducted using superficial dorsal horn neurons at the depths of 20 to 150 µm from the surface. Unit signals were acquired using an amplifier (EX1; Dagan Corporation, Minneapolis, MN, USA). The data were digitized with an analog-to-digital converter (Digidata 1400A; Molecular Devices, Union City, CA, USA) and analyzed with Clampfit (version 10.2; Molecular Devices, Union City, CA, USA). To determine the site of stimulation, we searched for sites where tactile stimuli on the skin (debrided cotton) or unpleasant plucking stimuli (forceps) caused a neural response. For mechanical stimulation, the skin was bent with thin vFFs, and bending forces of 5.88, 9.8, 13.72, 39.2, 58.8, 78.4, 147, 255 mN (0.6, 1.0, 1.4, 4.0, 6.0, 8.0, 15.0, 26.0 g) were applied, respectively. Stimulation was applied for 10 s at the maximum response point of each receptive field in the ipsilateral hindlimb. For the lidocaine evaluation, 0.5% lidocaine (AstraZeneca Japan, Osaka, Japan) was administered at the vFF stimulation site.

### 4.4. Laser Irradiation

A semiconductor laser light source (ML6500 system; Modulight Corporation, Tampere, Finland) was used. The laser beam was guided from the laser source by an optical fiber (M28L05; Thorlabs Incorporated, Newton, NJ, USA) and collimated by the lens (SLB-15-30-PIR1; SIGMAKOKI Company, Limited, Tokyo, Japan). A steel aperture was used to shape the laser beam with a diameter of 1 cm. Laser power, irradiation time, and oscillation mode were controlled by the laser source software (ML6700 Controller; Modulight Corporation, Tampere, Finland). Laser power was measured with a power meter (Display; NOVAII, Sensor; 10A-1.1V; Ophir Japan Limited, Saitama, Japan). Laser conditions were set as shown in Table 1.

The laser directly irradiated the right sciatic nerve, which was exposed through a skin incision. For sham irradiation, the nerve was exposed, and no laser irradiation occurred.

### 4.5. Histopathological Evaluation

Both sciatic nerves were harvested from laser-irradiated or sham-irradiated rats and immersion-fixed in 4% paraformaldehyde. The specimens were prepared by paraffin embedding and HE staining.

### 4.6. Statistical Analysis

Prism ver.8.4.3 (Graph Pad Software Incorporated, San Diego, CA, USA) was used for statistical analysis. The data are presented as the means ± SEM. For statistical analyses, we performed one-way analysis of variance followed by Wilcoxon matched-pairs signed-rank test and Dunnett’s multiple comparison; *p* < 0.05 was considered to be significant. Statistical significance is defined as * *p* < 0.05 and ** *p* < 0.01.

## 5. Conclusions

We showed that the nociception-evoked neuronal firing recorded in lamina II of the spinal dorsal horn was inhibited by irradiation of 808 nm lasers on peripheral nerve axons, which are conduction pathways for nociception stimuli. This suggests that the activity of Aδ and/or C fibers transmitting nociceptive stimuli is inhibited by laser irradiation without damage. Further investigation is required to elucidate the mechanism by which Aδ and/or C fibers are inhibited by laser irradiation.

## Figures and Tables

**Table 1:** Laser parameters.

Wavelength	808 nm
Power	790 mW
Area	0.79 cm^2^
Power density	1 W/cm^2^
Irradiation time	180 s
Fluence	180 J/cm^2^
Mode	Continuous wave

## Data Availability

The original contributions presented in the study are included in the article, and further inquiries can be directed to the corresponding author.

## References

[1] Dompe C., Moncrieff L., Matys J., Grzech-Leśniak K., Kocherova I., Bryja A., Bruska M., Dominiak M., Mozdziak P., Skiba T.H.I. (2020). Photobiomodulation—Underlying Mechanism and Clinical Applications. J. Clin. Med..

[2] Cheng K., Martin L.F., Slepian M.J., Patwardhan A.M., Ibrahim M.M. (2021). Mechanisms and Pathways of Pain Photobiomodula-tion: A Narrative Review. J. Pain.

[3] Bjordal J.M., Johnson M.I., Lopes-Martins R.A., Bogen B., Chow R., Ljunggren A.E. (2007). Short-Term Efficacy of Physical Interven-tions in Osteoarthritic Knee Pain. A Systematic Review and Meta-Analysis of Randomised Placebo-Controlled Trials. BMC Musculoskelet. Disord..

[4] Chow R., Armati P., Laakso E.-L., Bjordal J.M., Baxter G.D. (2011). Inhibitory Effects of Laser Irradiation on Peripheral Mammalian Nerves and Relevance to Analgesic Effects: A Systematic Review. Photomed. Laser Surg..

[5] Chow R.T., Johnson M.I., Lopes-Martins R.A., Bjordal J.M. (2009). Efficacy of Low-Level Laser Therapy in the Management of Neck Pain: A Systematic Review and Meta-Analysis of Randomised Placebo or Active-Treatment Controlled Trials. Lancet.

[6] Gomes A.O., Martimbianco A.L.C., Junior A.B., Horliana A.C.R.T., da Silva T., Santos E.M., Fragoso Y.D., Fernandes K.P.S., Nammour S., Bussadori S.K. (2022). Photobiomodulation for the Treatment of Primary Headache: Systematic Review of Randomized Clinical Trials. Life.

[7] De Barros D.D., Catão J.S.d.S.B., Ferreira A.C.D., Simões T.M.S., Almeida R.d.A.C., Catão M.H.C.d.V. (2022). Low-Level Laser Therapy Is Effective in Controlling Postoperative Pain in Lower Third Molar Extractions: A Systematic Review and Me-ta-Analysis. Laser Med. Sci..

[8] Tripodi N., Feehan J., Husaric M., Sidiroglou F., Apostolopoulos V. (2021). The Effect of Low-Level Red and near-Infrared Photo-biomodulation on Pain and Function in Tendinopathy: A Systematic Review and Meta-Analysis of Randomized Control Trials. BMC Sport. Sci. Med. Rehabilit..

[9] Yang J., Mallory M.J., Wu Q., Bublitz S.E., Do A., Xiong D., Chen C.Y.Y., Dorsher P.T., Chon T.Y., Bauer B.A. (2020). The Safety of Laser Acupuncture: A Systematic Review. Med. Acupunct.

[10] Chow R.T., Armati P.J. (2016). Photobiomodulation: Implications for Anesthesia and Pain Relief. Photomed. Laser Surg..

[11] Hsieh Y., Chou L., Chang P., Yang C., Kao M., Hong C. (2012). Low-level Laser Therapy Alleviates Neuropathic Pain and Pro-motes Function Recovery in Rats with Chronic Constriction Injury: Possible Involvements in Hypoxia-inducible Factor 1α (HIF-1α). J. Comp. Neurol..

[12] Cidral-Filho F.J., Mazzardo-Martins L., Martins D.F., Santos A.R.S. (2014). Light-Emitting Diode Therapy Induces Analgesia in a Mouse Model of Postoperative Pain through Activation of Peripheral Opioid Receptors and the l-Arginine/Nitric Oxide Pathway. Laser Med. Sci..

[13] Chow R.T., David N.A., Armati P.J. (2007). 830 Nm Laser Irradiation Induces Varicosity Formation, Reduces Mitochondrial Membrane Potential and Blocks Fast Axonal Flow in Small and Medium Diameter Rat Dorsal Root Ganglion Neurons: Implications for the Analgesic Effects of 830 Nm Laser. J. Peripher. Nerv. Syst..

[14] Holanda V.M., Chavantes M.C., Wu X., Anders J.J. (2017). The Mechanistic Basis for Photobiomodulation Therapy of Neuropathic Pain by near Infrared Laser Light. Laser Surg. Med..

[15] De Sousa M.V.P., Kawakubo M., Ferraresi C., Kaippert B., Yoshimura E.M., Hamblin M.R. (2018). Pain Management Using Photo-biomodulation: Mechanisms, Location, and Repeatability Quantified by Pain Threshold and Neural Biomarkers in Mice. J. Biophotonics.

[16] Tsuchiya K., Kawatani M., Takeshige C., Sato T., Matsumoto I. (1993). Diode Laser Irradiation Selectively Diminishes Slow Com-ponent of Axonal Volleys to Dorsal Roots from the Saphenous Nerve in the Rat. Neurosci. Lett..

[17] Tsuchiya K., Kawatani M., Takeshige C., Matsumoto I. (1994). Laser Irradiation Abates Neuronal Responses to Nociceptive Stimu-lation of Rat-Paw Skin. Brain Res. Bull..

[18] Yan W., Chow R., Armati P.J. (2011). Inhibitory Effects of Visible 650-nm and Infrared 808-nm Laser Irradiation on Somatosensory and Compound Muscle Action Potentials in Rat Sciatic Nerve: Implications for Laser-induced Analgesia. J. Peripher. Nerv. Syst..

[19] Yoshimura M., Nishi S. (1993). Blind Patch-Clamp Recordings from Substantia Gelatinosa Neurons in Adult Rat Spinal Cord Slices: Pharmacological Properties of Synaptic Currents. Neuroscience.

[20] Shimoyama N., lijima K., Shimoyama M., Mizuguchi T. (1992). The Effects of Helium-Neon Laser on Formalin-Induced Activity of Dorsal Horn Neurons in the Rat. J. Clin Laser Med. Surg..

[21] Kono T., Kasai S., Sakamoto T., Mito M. (1993). Cord Dorsum Potentials Suppressed by Low Power Laser Irradiation on a Peripheral Nerve in the Cat. J. Clin. Laser Med. Surg..

[22] Uta D., Tsuboshima K., Nishijo H., Mizumura K., Taguchi T. (2021). Neuronal Sensitization and Synaptic Facilitation in the Super-ficial Dorsal Horn of a Rat Reserpine-Induced Pain Model. Neuroscience.

[23] Chow R., Yan W., Armati P. (2012). Electrophysiological Effects of Single Point Transcutaneous 650 and 808 Nm Laser Irradi-ation of Rat Sciatic Nerve: A Study of Relevance for Low-Level Laser Therapy and Laser Acupuncture. Photomed. Laser Surg..

[24] Ishibashi N., Shimoyama H., Kawase Y., Motohara S., Okayama T., Niwa D., Koyama J. (2018). Measurement of Light Penetration of Near-Infrared Laser at the Lumbosacral Nerves in Rats. Mech. Photobiomod..

[25] Liao C.-D., Tsauo J.-Y., Liou T.-H., Chen H.-C., Rau C.-L. (2016). Efficacy of Noninvasive Stellate Ganglion Blockade Performed Using Physical Agent Modalities in Patients with Sympathetic Hyperactivity-Associated Disorders: A Systematic Review and Meta-Analysis. PLoS ONE.

[26] Holanda V.M., Chavantes M.C., Silva D.F.T., de Holanda C.V.M., de Oliveira J.O., Wu X., Anders J.J. (2016). Photobiomodulation of the dorsal root ganglion for the treatment of low back pain: A pilot study. Laser. Surg. Med..

[27] Torp K.D., Metheny E., Simon L.V. (2022). Lidocaine Toxicity. [Updated 21 November 2022]. StatPearls.

[28] Cotler H.B., Chow R.T., Hamblin M.R., Carroll J. (2015). The Use of Low Level Laser Therapy (LLLT) For Musculoskeletal Pain. MOJ Orthop. Rheumatol..

[29] Derouiche S., Li T., Sakai Y., Uta D., Aoyagi S., Tominaga M. (2022). Inhibition of Transient Receptor Potential Vanilloid 1 and Transient Receptor Potential Ankyrin 1 by Mosquito and Mouse Saliva. Pain.

[30] Hara K., Harris R.A. (2002). The Anesthetic Mechanism of Urethane: The Effects on Neurotransmitter-Gated Ion Channels. Aneasth. Analg..

[31] Uta D., Oti T., Sakamoto T., Sakamoto H. (2021). In Vivo Electrophysiology of Peptidergic Neurons in Deep Layers of the Lumbar Spinal Cord after Optogenetic Stimulation of Hypothalamic Paraventricular Oxytocin Neurons in Rats. Int. J. Mol. Sci..

